# *Chlamydia trachomatis* and *Chlamydia pneumoniae* Interaction with the Host: Latest Advances and Future Prospective

**DOI:** 10.3390/microorganisms7050140

**Published:** 2019-05-16

**Authors:** Marisa Di Pietro, Simone Filardo, Silvio Romano, Rosa Sessa

**Affiliations:** 1 Department of Public Health and Infectious Diseases, Section of Microbiology, University of Rome “Sapienza”, 00185 Rome, Italy; simone.filardo@uniroma1.it (S.F.); rosa.sessa@uniroma1.it (R.S.); 2 Cardiology, Department of Life, Health and Environmental Sciences, University of L’Aquila, 67100 L’Aquila, Italy; silvio.romano@univaq.it

**Keywords:** *Chlamydia trachomatis*, *Chlamydia pneumoniae*, host-pathogen interaction

## Abstract

Research in *Chlamydia trachomatis* and *Chlamydia pneumoniae* has gained new traction due to recent advances in molecular biology, namely the widespread use of the metagenomic analysis and the development of a stable genomic transformation system, resulting in a better understanding of *Chlamydia* pathogenesis. *C. trachomatis*, the leading cause of bacterial sexually transmitted diseases, is responsible of cervicitis and urethritis, and *C. pneumoniae*, a widespread respiratory pathogen, has long been associated with several chronic inflammatory diseases with great impact on public health. The present review summarizes the current evidence regarding the complex interplay between *C. trachomatis* and host defense factors in the genital micro-environment as well as the key findings in chronic inflammatory diseases associated to *C. pneumoniae*.

## 1. Introduction

Currently, there is a renewed research interest in *Chlamydiae* that cause a broad spectrum of pathologies of varying severity in human, mainly *Chlamydia trachomatis* and *Chlamydia pneumoniae* [1,2]. Advances in molecular biology and, in particular, the recent advent of metagenomic analysis as well as the development of a stable genomic transformation system in *Chlamydiae* have significantly contributed to expanding our understanding of *Chlamydia* pathogenesis [3,4,5].

*C. trachomatis* is the leading cause of bacterial sexually transmitted diseases with 127 million new cases per year, according to the most recent World Health Organization estimates [6]. In fact, *C. trachomatis* is well known as common cause of cervicitis and urethritis; however, most genital infections in women are asymptomatic and if untreated can lead to severe reproductive sequelae including pelvic inflammatory disease, ectopic pregnancy, obstructive infertility as well as miscarriages and preterm birth [7,8]. Furthermore, *C. trachomatis* infection can also be transmitted to infants following the direct contact with infective cervical secretions during delivery, resulting in neonatal conjunctivitis and pneumonitis [1,7,8]. Lastly, there is evidence that *C. trachomatis* infection increases the risk of acquiring and transmitting human immunodeficiency virus by 3 to 4 times and, more recently, it has been associated with Human Papillomavirus related-cervical cancer [9,10].

*C. pneumoniae* is a widespread respiratory pathogen responsible for sinusitis, pharyngitis, and pneumonia and its transmission occurs via the aerial route [11]. A peculiar feature of *C. pneumoniae* is its ability to systematically disseminate from the lungs through peripheral blood mononuclear cells and to localize in several extra-pulmonary tissues including arteries, joints, bone and the central nervous system [12,13,14,15,16,17]. Indeed, *C. pneumoniae* has long been associated with several chronic inflammatory diseases with great impact on public health, mainly atherosclerosis, Alzheimer’s Disease, and inflammatory arthritis [17,18,19,20,21,22,23,24]. This is unsurprising since *C. pneumoniae* has been shown to multiply in all cell types involved in the pathogenesis of these conditions, including monocytes/macrophages, synovial cells, vascular endothelial and smooth muscle cells (VSMCs), microglial cells, astrocytes and neurons [17,22,23,24].

The present review summarizes the current evidence regarding the complex interplay between *C. trachomatis* and host defense factors in the genital micro-environment as well as the key findings in chronic inflammatory diseases associated to *C. pneumoniae*.

## 2. *Chlamydiae* Developmental Cycle

*C. trachomatis* and *C. pneumoniae* are Gram-negative obligate intracellular bacteria with a peculiar developmental cycle alternating between two morphologically and functionally distinct forms: the elementary body (EB) and the reticulate body (RB) [25]. The EB is the small (200 nm), extracellular infectious form, classically considered as metabolically inactive, although recent studies have shown that EBs maintain protein translation capabilities, whereas the RB is the large (800 nm), intracellular, metabolically active replicative form [25,26,27].

**Figure 1:**
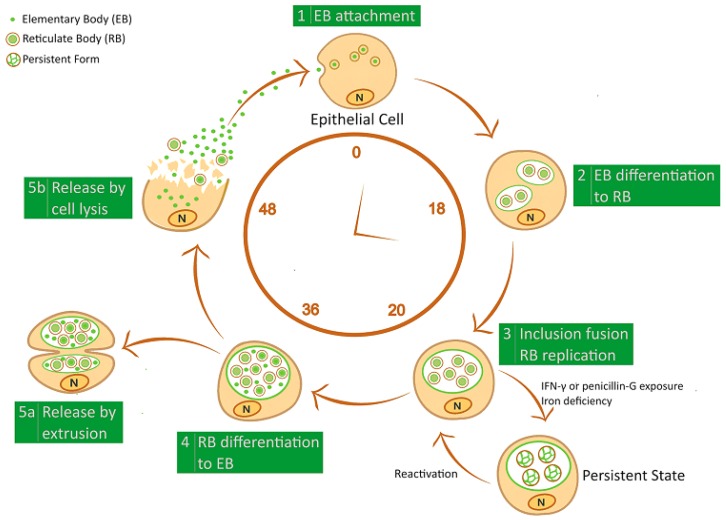
Schematic representation of *Chlamydiae* developmental cycle. Infectious elementary body (EB) enters into the host-cell and transforms in the replicative reticulate body (RB); RB re-differentiates into EB, which is released from the host-cell by inclusion extrusion or cell lysis. Exposure to IFN-γ and penicillin G or iron depletion induce *Chlamydiae* to generate a non-infectious persistent form.

The developmental cycle begins when EBs attach and enter the host cell by endocytosis (Figure 1). It is thought that the interaction of EBs with the host cell occurs in a two-step process involving a reversible interaction mediated by heparin-sulphate proteoglycans followed by irreversible binding to a wide range of host receptors: mannose receptor, epidermal growth factor receptor, ephrin receptor A2, and β1 integrin [28,29]. Soon after the attachment to host cell, EBs are internalized and confined to a vacuole termed the inclusion, through a process requiring the secretion of Type III secretion system (T3SS) effector proteins (e.g., Incs), as well as other chlamydial proteins, like the chlamydia protease-like activity factor (CPAF) and the high temperature requirement A protein (HtrA) [28,29]. Chlamydial Incs, inserted into the inclusion membrane, allow the escape of EB endosome from the endocytic-lysosomal pathway [30,31]. CPAF, a serine protease, plays a role in maintaining the integrity of the inclusion and promotes virulence by interfering with several host antimicrobial pathways such as apoptosis and complement system [32,33]. Lastly, HtrA, a serine protease as well, has been recognized as a critical factor for intracellular survival of *Chlamydiae* [34].

Within the inclusion, EBs then differentiate to RBs, which replicate by binary fission within 24 h post-infection and, as the inclusion expands, RBs begin to transition back to EBs in an asynchronous process. At the end of the developmental cycle, the inclusion occupies most of the host cell’s cytoplasm and, after approximately 48–72 h, the EBs are finally released from the host cell by inclusion extrusion or cell lysis. Thereafter, a multitude of infectious EBs spreads and infects neighboring epithelial cells, perpetuating the infectious process [25].

However, under stressful conditions, *Chlamydiae* halted the production of infectious EBs leading to viable but non-infectious forms characterized by continued synthesis of unprocessed 16S rRNA and genomic replication. These persistent forms are able to remain for a long-time in the host cell and are frequently associated with the presence of enlarged and morphologically aberrant RBs that retain their ability to resume the normal developmental cycle when the inducer is removed [35,36].

Several factors have been demonstrated to induce persistent forms via in vitro models including the exposure to interferon gamma (IFN)-γ or antibiotics (e.g., penicillin and amoxicillin), and nutrient deprivation (e.g., essential amino-acids or iron) [35,36,37,38,39,40]. Furthermore, it has been evidenced that coinfection with Herpes Simplex Virus type 2 or *Toxoplasma gondii* induces *C. trachomatis* persistent forms [36,41,42].

Importantly, these stress conditions may also occur in vivo [43,44] and, most notably, chlamydial persistence is supported by numerous observations of chlamydial aberrant forms in several tissues [45,46].

A relevant feature of chlamydial persistent form is its resistance to first line antibiotics towards *Chlamydiae* including and azithromycin [44,47]. This aspect alongside their ability to evade the host immune response may favor the long-term survival of *Chlamydiae* within tissues, resulting in a chronic inflammatory state and the subsequent tissue damage [48].

## 3. Genomic Modification Approaches in *Chlamydiae*

In the field of *Chlamydia* research, the insertion of exogenous DNA has always represented a big challenge [49,50] and, only recently, a reliable and robust transformation system has been developed for *C. trachomatis* [5], becoming the preferred technique for its recombination. This genomic transformation system has been utilized for the ectopic expression of reporter proteins conferring fluorescence to *C. trachomatis*, to either visualize live bacteria or investigate the localization of tagged-proteins during the chlamydial developmental cycle, like Incs [51,52]. At first, only promoters for the constitutive expression of target genes were used, then inducible promoter systems for conditional gene expression were developed, like the Tet System [53].

However, methods for the deletion or repression of a target gene, highly needed for investigating the molecular function of gene products, are still in development. Different approaches based on chemical mutagenesis or the TargeTron System were attempted [54,55,56,57], but both tools had important limitations. These were recently overcome, for the most part, by the development of a fluorescence-reported allelic exchange mutagenesis (FRAEM) system, through the engineering of a suicide vector by Mueller et al., 2016 [58], although, further studies will be necessary to optimize and validate these innovative techniques.

To be noted, these molecular tools were developed and applied to different strains of *C. trachomatis*, and, until recently, none of them were suitable for the genetic manipulation of *C. pneumoniae*. However, the *C. pneumoniae* plasmid shuttle vector, engineered by Shima et al. [59], enabled the generation of stable transformants in isolates of *C. pneumoniae*, providing the first tool for the transformation of this pathogen.

## 4. *C. trachomatis* Interaction with Host Defense Factors

The female genital tract is an ecological niche where several aerobe and anaerobe microorganisms coexist in a dynamic balance [4,5,60]. The homeostasis of the genital ecosystem results from complex interactions and synergies among the host and the resident microorganisms [3,4]. Changes in the structure and composition of this microbial ecosystem are influenced by several factors like age, menarche, pregnancy, infections, hormonal contraception, and sexual activity [61].

It is generally accepted that a healthy genital microbiota is typically dominated by *Lactobacillus* species, but other microorganisms, such as *Staphylococcus*, *Ureaplasma*, *Corynebacterium*, *Streptococcus*, *Peptostreptococcus*, *Gardnerella*, *Bacteroides*, *Mycoplasma*, *Enterococcus*, *Escherichia*, *Veillonella*, *Bifidobacterium* and *Candida* can be present in much lower amounts [4,5].

Eventually, the depletion of lactobacilli and the overgrowth of *Gardnerella vaginalis* or *Candida* spp. is known to lead to numerous clinical conditions, like bacterial vaginosis and candidiasis potentially associated to biofilm formation [62,63,64]. In our recent study, *C. trachomatis* was demonstrated to survive within the biofilm produced by *Candida albicans* or *G. vaginalis*, retaining its infectious properties [65]. This evidence is of clinical relevance since the biofilm, known as a protective niche, might favor *C. trachomatis* evasion of the host immune system and reduce its antibiotic susceptibility.

*Lactobacillus* spp. are the main host defense factor against pathogens, like *C. trachomatis*, within the cervico-vaginal ecosystem; in fact, they are able to limit the growth of genital pathogens through different mechanisms, such as competitive exclusion, anti-microbial compound production (lactic acid, hydrogen peroxide, defensins, etc.), the immune system activation as well as the maintenance of a low vaginal pH [66,67,68].

According to Gong et al. [69], lactic acid and, hence, a low pH, were demonstrated as essential for the anti-chlamydial activity of predominant *Lactobacillus* species in the cervico-vaginal microbiota. Since then, several studies reported the ability of different vaginal *Lactobacillus* strains such as *Lactobacillus brevis* or *Lactobacillus crispatus* to strongly inhibit early phases of *C. trachomatis* infection as well as its intracellular replication. In particular, several potential mechanisms interfering with *C. trachomatis* adhesion to host cell have been described, including the increased production of lactate and consumption of glucose, the co-aggregation with EBs, the changes in lipid composition of the cell membrane as well as the modulation of the α5 integrin subunit [70,71,72]. As a further defense mechanism, *L. brevis* has been demonstrated to inhibit the development of *C. trachomatis* persistent forms induced by HSV-2 coinfection [70]. Finally, *Lactobacillus* species may also protect the genital tract via immunomodulatory mechanisms. Specifically, in *C. trachomatis*-infected cervical epithelial cells and macrophages, *L. crispatus* has been shown to down-regulate the production of the cytokines frequently associated to tissue damage, like interleukin (IL)-6, IL-8 and tumor necrosis factor (TNF)-α, and, at the same time, to up-regulate IL-10 expression, an anti-inflammatory cytokine [73].

Alongside the resident lactobacilli, the female genital tract possesses other defense systems known to protect against *C. trachomatis*. Amongst them, lactoferrin, an 80-kDa multifunctional cationic glycoprotein belonging to the transferrin family, has acquired increasing interest for its marked anti-inflammatory and anti-chlamydial activities [74,75,76,77,78]. In fact, lactoferrin is released in the cervico-vaginal fluid by mucosal epithelial cells and neutrophils following *C. trachomatis* infection, as evidenced by higher levels of lactoferrin in infected rather than in healthy women [79,80,81].

Particularly interesting, in a clinical scenario, is the observation that the combination of lactoferrin and *L. brevis* is the most effective in inhibiting the early phases of *C. trachomatis* infection of cervical epithelial cells and in decreasing inflammatory cytokine synthesis, suggesting an additive effect of both host defense factors [77].

In addition to lactoferrin, other host defense peptides including defensins and cathelicidins, released in the cervico-vaginal fluid from genital epithelial cell and/or recruited neutrophils, have been demonstrated to inhibit *C. trachomatis* infection by inactivating EBs or by preventing their entry into the host cell as well as their intracellular growth [82,83,84,85]. However, it was recently demonstrated that cathelicidin LL-37 was degraded by the CPAF secreted by *C. trachomatis* [86]. Such an observation is of pathological significance since it describes one of the potential mechanisms by which *C. trachomatis* infection can spread into the upper genital tract and, hence, result in severe reproductive sequelae.

### Genital Microbiota Characterization by Metagenomic Analysis

Over last few years, culture-independent high-resolution techniques based on the analysis of 16s ribosomal RNA gene sequences have contributed to expanding our knowledge on the composition of the genital microbiota, leading to its classification into five community state types (CSTs I-V) [4,87,88,89,90].

In healthy reproductive women, the cervico-vaginal mucosa is mostly populated by *L. crispatus* (CST I) and *L. gasseri* (CST II) dominated microbiota [4,5,68,89]. In fact, *L. crispatus*, as well as *L. gasseri*, are known to produce D-lactic acid, bacteriocins and other anti-microbial compounds that provide protection against genital pathogens [68,89]. By contrast, women with *C. trachomatis* or *C. trachomatis*/HPV coinfection possess a genital microbiota dominated by *L. iners* (CSTs III) or by different anaerobic bacterial species (CST-IV) [81,91,92,93,94,95,96,97,98,99]. On this regard, it has been demonstrated that some anaerobes, like *Prevotella* ssp., frequently observed in dysbiosis conditions, are able to produce indole allowing *C. trachomatis* to elude the IFN-γ-mediated host immune response [98,100,101]. At the same time, it is very likely that the latter generates chlamydial persistent forms which in presence of indole producing bacteria may revert to active developmental cycle resulting in recurrent infection.

More recently, for the first time, a specific cervical bacterial network including *G. vaginalis*, *Prevotella amnii*, *Prevotella buccalis*, *Prevotella timonensis*, *Aerococcus christensenii* and *Variovorax guangxiensis* has been proposed as a potential biomarker for *C. trachomatis* infection. This interesting data may add up valuable information to the ongoing research on the cervical microbiota associated to *C. trachomatis* infection and may help to identify women at risk of infection [81]. In the future, it will be important to perform longitudinal studies in order to monitor the temporal dynamics of the cervical microbiota during *C. trachomatis* infection.

## 5. *C. pneumoniae* and Chronic Inflammatory Diseases

Over the past decades, a growing number of studies have focused on the involvement of *C. pneumoniae* in chronic inflammatory diseases, mainly atherosclerotic cardiovascular diseases, Alzheimer’s Disease and reactive arthritis (ReA) [17,18,19,20,21,22,23,24]. Recently, the role of the infectious burden, including more infectious agents alongside *C. pneumoniae*, acquired importance as a novel view for the etiopathogenesis of these diseases [102,103,104].

### 5.1. Atherosclerotic Cardiovascular Diseases

Atherosclerotic cardiovascular disease (CVD) is the leading cause of death worldwide with over 17 million deaths per year [105] and the main pathological process underlying this disease is the atherosclerosis.

The relationship between *C. pneumoniae* and CVDs was been first suggested in 1988, by Saikku et al. [106]. Since then, accumulating evidence has supported the involvement of *C. pneumoniae* in the pathogenesis of CVDs, including seroepidemiological studies, the detection of *C. pneumoniae* DNA in the atherosclerotic plaque and the isolation of viable bacteria from the atheroma [17,107,108,109,110,111,112,113]. Stronger evidence came from in vivo studies demonstrating a causative role of *C. pneumoniae* in the pathogenesis of the atherosclerosis. Specifically, *C. pneumoniae* has been shown to promote endothelial dysfunction in normolipidemic and hyperlipidemic animal models as well as to accelerate the progression of atherosclerotic lesion in hyperlipidemic animals [17,114,115].

Particularly important are the experimental studies that highlighted the molecular mechanisms linking oxidative stress and inflammation to *C. pneumoniae*-mediated atherosclerosis. Indeed, numerous are the evidence demonstrating the ability of *C. pneumoniae* to induce oxidative stress and, hence, contribute to the early as well as the late stages of the atherosclerotic process by promoting endothelial dysfunction, foam cell formation and platelet activation [116,117]. Specifically, *C. pneumoniae* infection of vascular cells has been shown to upregulate multiple enzymatic systems capable of producing reactive oxygen species, including NADPH oxidase, lipoxygenase and cyclooxygenase as previously described [117]. Recently, *C. pneumoniae* has also been found to interfere with endothelial nitric oxide (NO) synthase impairing NO production and, hence, leading to vascular dysfunction [118].

Concerning inflammatory pathways, several studies have demonstrated the ability of *C. pneumoniae* to induce in macrophages, platelets, endothelial cells and VSMCs an increased production of pro-inflammatory cytokines and adhesion molecules, such as IL-6, IFN-γ, TNF-α, intercellular adhesion molecule-1 and vascular cell adhesion molecule-1, all responsible for the initiation, progression and destabilization of the atherosclerotic plaque [119,120].

More recently, further studies have highlighted that inflammatory and immune mechanisms activated by *C. pneumoniae* alongside dyslipidemia may play a role in the development and progression of the atherosclerotic plaque. For example, Turmurkhuu et al. [121] found that *C. pneumoniae* was able to activate Nod-like receptor family pyrin domain-containing 3 (NLRP3) inflammasome with subsequent increase of IL-1β in macrophages resulting in accumulation of intracellular cholesterol and foam cell formation. Again, Chen et al. [122] observed that *C. pneumoniae* and lipids engaged the same innate immune signaling pathways (Toll Like receptor-4/myeloid differentiation primary response 88), accelerating the atherosclerotic process.

In addition to the vascular inflammation, there is also the evidence that *C. pneumoniae* may contribute to CVDs via the systemic inflammation [123]. However, despite the numerous evidence supporting the *C. pneumoniae* involvement in the pathogenesis of atherosclerosis, its causative role still needs to be assessed due to the failure of clinical antibiotic trials [124].

Of particular relevance is the recent observation that the infectious burden, including *C. pneumoniae*, may be involved in the development of atherosclerosis and the subsequent cardiovascular events. Potentially, an individual may be exposed to more microbial agents during his lifetime rather than to a single pathogen, since more than half of the world population is seropositive to, for example, *C. pneumoniae*, *Helicobacter pylori* or human cytomegalovirus [17,125,126]. Zhu et al. [127] were the first to show the association between a high risk of coronary artery disease and *C. pneumoniae* alongside other bacterial and/or viral pathogens, and, thereupon, further studies have contributed to strengthen this interesting hypothesis [102,128].

More importantly, the novel idea of a vascular microbiome involved in the development of atherosclerosis has been recently suggested by the detection of a microbiota in the atherosclerotic plaque, hypothesizing the oral cavity and/or the gut as the bacterial source [129,130,131,132].

### 5.2. Alzheimer’s Disease

Alzheimer’s Disease (AD) is an inflammatory brain disease that affects more 45 million people worldwide and is associated with a combination of genetic and environmental factors, leading to inflammation of the brain, neuronal cell death and progressive dementia [133].

The first evidence that *C. pneumoniae* may be involved in AD was reported in 1998 by Balin et al. [134] through the detection of *C. pneumoniae* in brain tissue from patients with late-onset dementia and, later on, by Dreses-Werringloer et al. [135] through the isolation of viable microorganism from post-mortem brain tissue samples of AD patients; since then, a growing number of studies have been published, although with controversial results. However, a recent meta-analysis study confirmed a positive association between *C. pneumoniae* and AD [20,24,136,137,138].

Further evidence on the involvement of *C. pneumoniae* in the pathogenesis of AD came from studies showing the ability of this pathogen to disseminate to the brain via the olfactory system or following a vascular infection and, hence, to induce or accelerate the formation of amyloid deposits, a pathological feature typically observed in AD patients [24,139,140,141].

Similar to *C. pneumoniae*-associated atherosclerosis, inflammation seems to play a role in AD pathogenesis as well. Indeed, it has been demonstrated the ability of *C. pneumoniae* to elicit the production of IL-6 and TNF-α, responsible for neuronal death, in microglial cells and astrocytes, and the synthesis of IL-1β and IL-8, known to elicit neurodegeneration in AD via the activation of nitric oxide synthase, in persistently infected monocytes [24].

Several other mechanisms by which *C. pneumoniae* may contribute to the development and progression of AD have also been documented, including the potential interaction with host genetic factors, namely the ApoE_ɛ_4 isoform, a known risk factor for the development of late–onset dementia [24], and the inhibition of apoptosis in neuroblastoma cells, leading to a long-term infection [142].

More recently, the involvement of the infectious burden has also been proposed in the pathogenesis of AD by Bu et al. [104], through the detection of more viral and/or bacterial pathogens, including *C. pneumoniae*, in AD patients, evidencing a marked inflammatory state. Interestingly, it has been demonstrated that *C. pneumoniae* and other pathogens expressed proteins with marked homology to amyloid-β (Aβ) and amyloid precursor protein (APP), suggesting that infections may trigger autoantibodies that cross-reacted with membrane bound APP and caused synaptic and neuronal dysfunction and subsequent cognitive decline [104,143].

Despite all the evidence, in the only clinical trial, the antibiotic treatment had some beneficial effects on the cognitive symptoms in AD patients, but it was ineffective against *C. pneumoniae* [144].

### 5.3. Reactive Arthritis

Reactive arthritis (ReA), an inflammatory syndrome that arises during or soon after bacterial infections occurring elsewhere in the body and classically related to *C. trachomatis*, has also been associated to *C. pneumoniae* in the last two decades [21,22].

To date, evidence for *C. pneumoniae* involvement in the development of ReA is exclusively based on the detection of chlamydial nucleic acid from synovial fluid or tissue in patients with ReA [145,146,147,148,149,150,151,152,153,154]. Surprisingly, prospective epidemiological studies estimated a much lower occurrence of ReA following *C. pneumoniae* (2.2%) as compared to its seroprevalence in the general population (80–90%) [148,149,155]. Several factors may explain this apparent disconnection, including the spontaneous resolution of most ReA, the subtle presentation in women and the lack of standardized diagnostic criteria, leading to an underestimation of the impact of ReA following *C. pneumoniae* infection [22].

In the last decade, the coinfection of *C. pneumoniae* with *C. trachomatis* in the synovial fluid of ReA patients has also been evidenced, suggesting the possibility that *C. pneumoniae* might act synergistically with *C. trachomatis* in the etiopathogenesis of this disease via the development of chronic inflammation in the joint [156,157]. Thereupon, the hypothesis that the infectious burden, including *C. pneumoniae*, might be involved in the etiopathogenesis of ReA has acquired importance, suggesting the possibility that multiple infections acted synergistically, increasing the risk of developing ReA [152,154,157].

## 6. Conclusions and Future Prospective

Important advances in the molecular mechanisms of pathogenicity as well as in the interaction with the host have been achieved in the field of *C. trachomatis* and *C. pneumoniae* research, although much more remains to be done.

Concerning *C. trachomatis*, an interesting finding is the survival of this pathogen within biofilm generated by resident microorganisms of the genital ecosystem [65]; this novel evidence is worthy of further investigation since the biofilm is frequently found on intrauterine devices and may contribute to *C. trachomatis* transmission as well as dissemination to the upper genital tract [158,159].

Interestingly, the recent characterization of the cervico-vaginal microbiota associated to *C. trachomatis* infection as well as the anti-chlamydial activity of host defense peptides [77,84,99] will be helpful to develop novel prevention or treatment strategies. In this regard, of pathophysiological and clinical relevance will be the discovery of novel mechanisms underlying the anti-*C. trachomatis* activity of the host defense factors, like *Lactobacillus* spp. or lactoferrin. For example, it will be important to clarify how *Lactobacillus* spp. limited *C. trachomatis* intracellular replication or how lactoferrin impaired chlamydial invasion.

Differently from *C. trachomatis*, research on *C. pneumoniae* in the field of chronic inflammatory diseases did not undergo significant development, due to difficulties in isolating and culturing *C. pneumoniae* as well as to the multifactorial etiology of these pathological conditions. However, in recent times, the involvement of the infectious burden [102,103,104], including *C. pneumoniae*, in the etiopathogenesis of chronic inflammatory diseases opened the way to further approaches.

An important issue that remains to be solved for both *C. pneumoniae* and *C. trachomatis* is the persistence state into host cell. In fact, *Chlamydiae* persistence might be one explanation for the failure of clinical trials in *C. pneumoniae*-associated chronic inflammatory diseases as well as in *C. trachomatis* recurrent infections [132,160,161].

For years, research on *Chlamydiae* persistent forms has focused on identifying a distinct transcriptional and protein profile, such as the up-regulation or the down-regulation of the genes, involved in RB division and/or differentiation into EBs [35,162], as well as on the potential survival strategies, such as the production of membrane vesicles [163], an alternative protein delivery system in host cell. However, despite all the efforts, the identification of a common persistence marker during chlamydial infection is still missing.

In the future, the application of the recently engineered transformation system for the insertion of foreign DNA sequences in *C. trachomatis* and *C. pneumoniae* will contribute to expanding our knowledge on *Chlamydiae* pathogenesis. In particular, it will allow us to precisely characterize the temporal dynamics, role and functions of the genes expressed during the different phases of chlamydial developmental cycle, uncovering, for example, the elusive mechanisms underlying the generation of persistent forms. Furthermore, these molecular tools will be needed to decipher the function of essential genes involved in the host cell interaction, providing, for example, novel targets for the development of an effective *Chlamydia* vaccine.
